# Lateral violence and its relationship with intention to quit among emergency department nurses at Ardabil Medical Training Centers

**DOI:** 10.1186/s12912-026-05211-9

**Published:** 2026-08-07

**Authors:** Mansooreh Karimollahi, Masoumeh Aghamohammadi, Farideh SHahrivar

**Affiliations:** Department of Nursing, School of Nursing and Midwifery, Ardabil University of Medical Sciences, Ardabil, Iran

**Keywords:** Lateral violence, Workplace violence, Emergency nurses, Turnover intention

## Abstract

**Purpose:**

Lateral violence among nurses is a critical workplace issue that threatens nurse well-being, patient safety, and staff retention. This study aimed to examine the prevalence of lateral violence and its relationship with intention to quit among emergency department nurses.

**Methods:**

This cross-sectional descriptive–correlational study was conducted in 2024 among 243 emergency department nurses working in educational medical centers affiliated with Ardabil University of Medical Sciences. A census sampling method was employed to include all eligible participants. Data were collected using a demographic questionnaire, a validated lateral violence questionnaire, and the intention-to-quit scale developed by Jung and Yoon. Data analysis was performed using descriptive statistics, Pearson correlation coefficients, t-tests, ANOVA, and linear regression analysis in SPSS version 25.

**Results:**

More than 60.4% of nurses reported experiencing or witnessing lateral violence at least once per month. Ignoring colleagues’ workload and harsh criticism without listening were the most frequently reported behaviors. The mean intention-to-quit score was 12.95 ± 4.49, indicating a moderate level of turnover intention. Significant positive correlations were found between exposure to lateral violence, failure to report violent behaviors, and intention to quit (*p* < 0.01).

**Conclusions:**

Lateral violence is prevalent among emergency department nurses and is significantly associated with intention to quit, particularly when violent behaviors are not reported. Nurse managers should implement supportive reporting systems and interventions to reduce lateral violence, which may contribute to improving nurse retention.

**Clinical trial number:**

Not applicable.

## Introduction

Workplace violence is recognized as a major occupational hazard in healthcare settings. The World Health Organization defines violence as the intentional use of physical force or power, threatened or actual, that results in or is likely to result in injury, psychological harm, deprivation, or death [1]. Among healthcare professionals, nurses are particularly vulnerable due to their continuous patient contact and high-stress work environments.

Lateral violence, also referred to as horizontal violence, is a form of workplace violence occurring between colleagues at the same organizational level [2]. It encompasses behaviors such as gossiping, belittling, ignoring, unfair criticism, and social isolation [3]. In nursing literature, this concept is often used interchangeably with terms such as horizontal violence [4], mobbing [5], bullying [6], and incivility [7]. Lateral violence can be better understood through Johnson’s Ecological Model of Workplace Violence, which is derived from Bronfenbrenner’s ecological perspective. This model explains workplace violence as the result of interactions across multiple levels, including immediate interpersonal relationships, professional interactions, organizational structures, and broader sociocultural norms. From this perspective, lateral violence among nurses is not merely an individual behavior, but a multifaceted workplace phenomenon shaped by the clinical environment, team dynamics, and institutional context [8]. Although often subtle and covert, lateral violence can persist over time and lead to psychological distress, reduced job satisfaction, and impaired professional functioning. Within the framework of Johnson’s Ecological Model, repeated exposure to lateral violence—occurring primarily at the interpersonal level—can damage the professional climate, leading to psychological distress and a sense of isolation among nurses. At the organizational level, when such behaviors are normalized or remain unaddressed due to organizational silence, nurses exhibit a higher reluctance to report these incidents. Ultimately, the combination of unresolved peer-to-peer hostility, lack of organizational support, and underreporting behaviors cumulatively increase their intention to quit, driving them to seek safer work environments.

In the Iranian healthcare context, workplace violence remains an important concern for nursing staff. Previous Iranian studies have shown that nurses are exposed not only to violence from patients and attendants, but also to lateral violence from colleagues. For example, a study in Mazandaran reported that 68.3% of nurses had experienced violent behaviors from fellow nurses [9], while a study conducted in Urmia during the COVID-19 pandemic reported a lateral violence prevalence of 45.38% [10]. Emergency departments may be particularly vulnerable to such behaviors because of heavy workload, stressful clinical conditions, rapid decision-making, and frequent exposure to crisis situations.

Evidence suggests that lateral violence compromises patient safety by increasing the risk of communication breakdowns, medication errors, and adverse events [11]. Moreover, exposure to lateral violence is a strong predictor of nurses’ intention to quit, contributing to workforce instability and increased organizational costs [12].

Despite the growing body of evidence in Iran, important gaps remain. Existing Iranian studies are limited in number and have not adequately addressed the specific context of emergency nurses in Ardabil. Moreover, although some studies have documented the prevalence of lateral violence, less is known about its relationship with nurses’ intention to quit in this clinical setting. Therefore, further evidence is needed to clarify the extent of lateral violence and its occupational implications among emergency nurses in Ardabil.

Accordingly, this study sought to examine the prevalence of lateral violence and its association with the intention to quit among emergency department nurses at Ardabil Medical Training Centers. The study was guided by the following research questions: (1) What is the prevalence of lateral violence among emergency department nurses in Ardabil? and (2) Is lateral violence associated with the intention to quit among these nurses?

## Methods

### Design and participants

#### This cross-sectional

A descriptive–correlational study was conducted in 2024 among emergency department nurses working in educational medical centers affiliated with Ardabil University of Medical Sciences. The eligible population consisted of 243 nurses working in the emergency departments of the selected centers during the study period. Using a census sampling method, all 243 eligible nurses were invited to participate, and all completed the questionnaires, resulting in a 100% response rate. Data were collected through face-to-face, self-administered questionnaires. Regarding instrument translation, we implemented a rigorous forward-backward translation process and conducted pilot testing to ensure cultural and linguistic equivalence of the tools.

The inclusion criteria were having at least 12 months of emergency department work experience and willingness to participate (informed consent). We did not include mental health screening as an exclusion criterion to avoid potential bias from self-reporting. Exclusion criteria included incomplete questionnaires or refusal to participate; however, no participants were excluded.

#### Instruments

Demographic questionnaire: Collected data on age, gender, marital status, education level, organizational position, work experience, emergency department experience, and shift type.

Lateral violence questionnaire: A composite instrument adapted from Dumont et al. [13], Khalil [14], and Wilson et al. [15]. consisting of 26 items across five domains: exposure to violence, perpetrators, personal impact, effects on quality of care, and reporting behaviors. Content validity was confirmed (CVI = 0.90; CVR = 0.90). The items were selected based on their conceptual relevance to the study aim and to the multidimensional nature of lateral violence as a workplace phenomenon. Because this instrument was used as a descriptive, item-based composite measure rather than as a single summative scale, the items were analyzed at the domain and item levels, and no total score was calculated. Given the heterogeneous response formats of the questionnaire, internal consistency testing such as Cronbach’s alpha was not considered appropriate.

Internal consistency metrics such as Cronbach’s alpha were not applied because the instrument was not used as a single summative scale. Its reliability was supported through the use of established source instruments and content validity assessment.

Thus, the questionnaire was used to describe multiple aspects related to lateral violence rather than to generate a single overall lateral violence score.

##### Intention-to-quit questionnaire

Developed by Jung and Yoon (2013) ; [16], consisting of four items rated on a 5-point Likert scale, from 1 (“strongly disagree”) to 5 (“strongly agree”). Previous studies reported acceptable reliability (Cronbach’s alpha > 0.70). The total score is calculated by summing the scores on all questions, resulting in a score range of 4 to 20, with higher scores indicating greater intention to leave.

The questionnaires were translated into Persian and back-translated by independent bilingual experts. Content equivalence was reviewed by the research team, and the final Persian version was pilot-tested for clarity.

#### Data analysis

Data were analyzed using SPSS version 25. Descriptive statistics including frequencies, percentages, means, and standard deviations, were used to summarize the demographic characteristics and study variables. Statistical significance was set at *p* < 0.05.

## Results

### Demographic characteristics

**Table 1:** Demographic characteristics of study participants (*N* = 243)

Variable	Category	Number (*N*)	percentage (%)
Age (years)	20–25	24	9.9
	26–30	71	29.2
	31–35	75	30.6
	36–40	54	22.2
	41–45	14	5.8
	Above 45	5	2.1
Gender	Woman	171	70.4
	Man	72	29.6
Marital Status	Single	92	37.4
	Married	151	62.1
Organizational Post	Nurse	238	97.9
	Superintendent	5	2.1
Education level	Bachelor	241	99.2
	Master’s degree	2	0.8
Work Experience (years)	5 and less	76	31.3
	6–10	82	33.7
	11–15	55	22.6
	16–20	23	9.5
	21–25	5	2.1
	Above 25	2	0.8
Work Experience in Emergency Department (years)	5 and less	149	61.3
	6–10	61	25.1
	11–15	29	11.9
	16–20	4	1.6
Shift Type	Rotating	230	94.7
	Fixed	13	5.3

Participants were predominantly female, married, and held a bachelor’s degree in nursing. Most worked rotating shifts and had 6–10 years of professional experience. Detailed demographic data are presented in (Table 1).

### Prevalence of lateral violence

**Table 2:** Responses to questions about lateral violence among emergency department nurses

Questions Over the past 12 months…	Never (%)	Once (%)	Rarely (%)	Monthly (%)	Weekly (%)	Daily (%)	Prevalence (≥ Monthly ) (%)
1. Observed non-responsible nursing colleagues exhibiting unprofessional behaviors	6.6	5.8	27.2	20.1	21.0	19.0	60.1
2. Observed supervisors exhibiting unprofessional behaviors	2.9	4.9	25.1	20.6	23.5	23.0	67.1
3. Witnessed severe criticism without hearing both sides	7.4	7.0	20.1	25.1	20.6	19.8	65.5
4. Witnessed humiliation or labeling of colleagues in front of others	13.6	8.2	19.8	23.5	20.1	14.8	58.4
5. Witnessed complaining to others instead of direct conflict resolution	8.6	5.4	23.9	25.1	19.3	17.7	62.1
6. Witnessed eye-rolling or raising eyebrows at colleagues	7.0	7.0	21.0	23.9	24.2	16.9	65
7. Witnessed disregard for the workload a colleague is struggling with	6.2	5.3	18.5	21.4	22.3	26.3	70
8. Felt discouraged due to lack of positive feedback	7.4	6.2	16.0	20.6	17.7	32.1	70.4
9. Remained silent about perceived wrongdoings due to fear of reprisal	19.8	4.9	31.7	19.3	9.9	14.4	43.6
10. Hesitated to ask questions due to fear of mockery	24.7	7.8	29.2	16.5	11.1	10.7	38.3
11. Left work feeling bad about myself due to interaction with colleagues	35.4	9.5	21.0	17.3	11.9	4.9	34.1
12. Experienced physical symptoms due to poor interaction with colleagues	29.2	11.5	19.8	22.2	9.9	7.4	39.5

Over the past 12 months, lateral violence was prevalent in the workplace; more than 60% of nurses reported witnessing such behaviors from colleagues, and over 67% reported similar actions from supervisors at least once per month.

The most frequently observed behaviors included ignoring a colleague’s workload (70.0%), harsh criticism without context (65.5%), and indirect conflict resolution (62.1%). Furthermore, a substantial proportion of nursing staff reported significant personal impacts, including reluctance to speak up due to fear of reprisal (43.6%) and the manifestation of physical symptoms stemming from negative workplace interactions (39.5%) (Table 2).

### Intention to quit

**Table 3:** Descriptive statistics of intention to quit score among emergency department nurses in 2024 (*N* = 243)

Variable	Minimum	Maximum	Mean	SD
Intention to quit	4.00	20.00	12.95	4.49

The mean intention-to-quit score was 12.95 ± 4.49. This score, being slightly above the scale’s midpoint of 12, indicates a moderate level of intention to quit among emergency department nurses (Table 3).

### Relationship between lateral violence and intention to quit

**Table 4:** Multiple regression analysis of predictors for intention to quit

Variable	B	SE	β	t	*p*	95% CI	*R* ^2^
(Constant)	1.89	0.31	-	6.10	< 0.001^***^	[1.28–2.50]	-
Lateral Violence	0.24	0.05	0.33	4.80	< 0.001^***^	[0.14–0.34]	0.23
Under-reporting	0.19	0.06	0.21	3.17	0.002^**^	[0.07–0.31]	0.23
Age	-0.04	0.06	-0.04	-0.67	0.504	[-0.016-0.08]	0.23
Gender	0.08	0.14	0.04	0.57	0.569	[-0.20-0.36]	0.23
Work Experience	-0.06	0.06	-0.06	-1.00	0.319	[-0.18-0.06]	0.23

**p < 0.01; ***p < 0.001

Multiple regression analysis revealed that the model accounted for 23% of the variance in the dependent variable. ‘Lateral violence’ (𝛽=0.33, 𝑝<0.001) and ‘underreporting’ (𝛽=0.21, 𝑝=0.002) were identified as significant positive predictors. In contrast, demographic variables, including age, gender, and work experience, did not significantly influence the dependent variable (𝑝>0.05) (Table 4).

## Discussion

This study revealed a high prevalence of lateral violence among emergency department nurses and confirmed its significant association with intention to quit [17]. Similarly, Zhan Yi’s study also reported a high prevalence of lateral violence among nurses and found that its occurrence varied across regions, suggesting that cultural context may influence its prevalence, although further evidence is needed to support this assumption [18].Both covert and overt forms of violence were commonly reported, which is consistent with the findings of previous studies [8, 9]. In contrast, Huiwen Xu et al. reported that nursing students experienced covert lateral violence more frequently than overt behaviors. Nevertheless, covert lateral violence is often overlooked by nursing managers, even though it may persist for a long time [19].

Ignoring colleagues’ workload emerged as the most prevalent form of covert violence, reflecting systemic issues such as staff shortages and high job demands. The strong association between underreporting and intention to quit highlights the importance of creating psychologically safe environments that encourage reporting without fear of retaliation [20]. Most nurses reported experiencing or witnessing behaviors such as harsh criticism without hearing both sides of the story, humiliating or labeling colleagues, negative nonverbal behaviors, and complaining instead of resolving conflicts directly. Being ignored by coworkers was also reported as a daily experience. These findings are consistent with the study by Peng et al., in which covert behaviors such as withholding information, ignoring others’ opinions, and spreading rumors, as well as some overt behaviors, were identified as the most common forms of lateral violence [21].

Nurses who were more frequently exposed to violence were less likely to report violent behaviors. In addition, a significant positive association was found between non-reporting of violent behaviors and intention to quit. Taken together, these findings suggest that nurses who are reluctant to report violent incidents may be at greater risk of exposure to lateral violence and may also be more likely to consider leaving their jobs. Similarly, Zhang et al. reported an association between lateral violence and intention to quit that was consistent with the findings of the present study [22].Consistent with the present study, Habibzadeh et al. also reported lateral violence as a strong predictor of intention to leave among clinical nurses, indicating that nurses who experienced higher levels of violence from their colleagues were more likely to intend to leave their jobs [10].

The results of this study indicate a remarkably high prevalence of lateral violence in the emergency department, with 92.6% of nurses witnessing ‘harsh criticism without hearing both sides’ at least once in the past year. Furthermore, 65.5% observed this behavior on a regular basis (monthly to daily). These results are consistent with previous research reporting a 59.2% overall exposure rate to lateral violence. However, the intensity of such behaviors appears significantly higher in our emergency-specific cohort. While the comparative study highlighted personal (51%) and social (37.3%) dimensions of violence, our data emphasizes a breakdown in professional communication and teamwork. This high frequency of one-sided criticism suggests a profound deficiency in conflict resolution mechanisms and psychological safety within the emergency setting, which likely acts as a precursor to broader organizational issues such as workplace isolation and turnover intention [23].

Our findings revealed that demographic and occupational factors, specifically gender and shift type, significantly influenced lateral violence experience and exposure among emergency nurses. Conversely, marital status, education level, age, and work tenure had no significant impact. This aligns with Vidal-Alves et al. [24], who challenge the traditional assumption that younger or less experienced nurses bear the brunt of workplace hostility, reporting instead higher rates among male nurses and those with 6–10 years of experience. These parallel findings suggest that lateral violence in emergency settings is shaped more by specific workplace dynamics and structural factors than by age or experience alone.

Considering the detrimental effects of sustained lateral violence on nurses’ well-being and organizational retention, these findings underscore the critical need for targeted interventions within nursing practice. The persistence of such behaviors, often overlooked or normalized, directly fuels nurses’ intention to quit by undermining their sense of psychological safety and increasing emotional exhaustion. Therefore, implementing robust strategies to prevent and address lateral violence is paramount for maintaining a stable and healthy nursing workforce.

## Limitations

Several limitations of this study should be acknowledged. First, the cross-sectional design precludes the establishment of causal relationships between lateral violence and turnover intention; therefore, results should be interpreted with caution. Second, the reliance on self-reported measures introduces the potential for self-report bias and social desirability bias, as participants may have provided responses that minimize the perceived prevalence of lateral violence or align with professional norms. Furthermore, the study was conducted in selected hospitals within a single region, which may limit the generalizability of the findings to broader contexts. Future research should employ longitudinal designs and multi-center approaches to validate these findings and establish temporal precedence.

## Conclusions

Lateral violence is a prevalent issue among emergency department nurses and is significantly associated with intention to quit, particularly when incidents are not reported. Organizational strategies aimed at reducing workplace violence and strengthening support systems are essential to improve nurse retention and mental well-being.

## Implications for nursing practice

Addressing lateral violence through organizational policies, psychological support, and leadership training may improve nurses’ mental health, job satisfaction, and retention, particularly in high-stress settings such as emergency departments.

## Data Availability

No datasets were generated or analysed during the current study.

## Abbreviations

ANOVA: Analysis of variance
SPSS: Statistical package for social science

## References

[1] Trivedi S, Bhardwaj H, Sahoo TK, Gupta S. Workplace Violence Against Nurses in Psychiatric Hospitals in Oman. Sultan Qaboos Univ Med J. 2024;24(2):194–202. 10.18295/squmj.7.2023.046 PMC11139374 38828244

[2] Bambi S, Foà C, De Felippis C, Lucchini A, Guazzini A, Rasero L. Workplace incivility, lateral violence and bullying among nurses. A review about their prevalence and related factors. Acta Bio Medica: Atenei Parmensis. 2018;89(Suppl 6):51. 10.23750/abm.v89i6-S.7461 PMC6357596 30038204

[3] Mao A, Tam HL, Cheong PL, Van IK. You need to get over the difficulties and stand up again—A qualitative inquiry into young nurses’ coping with lateral violence from the feminist perspective. Int J Environ Res Public Health. 2021;18(13):7167. 10.3390/ijerph18137167 PMC8297138 34281104

[4] Taylor R. Nurses’ perceptions of horizontal violence. Global qualitative Nurs Res. 2016;3:2333393616641002. 10.1177/2333393616641002 PMC5342647 28462334

[5] Yildirim A, Yildirim D. Mobbing in the workplace by peers and managers: mobbing experienced by nurses working in healthcare facilities in Turkey and its effect on nurses. J Clin Nurs. 2007;16(8):1444–53. 10.1111/j.1365-2702.2006.01814.x 17655532

[6] Quine L. Workplace bullying in nurses. J Health Psychol. 2001;6(1):73–84. 10.1177/135910530100600106 22049239

[7] Clark C. The dance of incivility in nursing education as described by nursing faculty and students. Adv Nurs Sci. 2008;31(4):E37–54. 10.1097/01.ANS.0000341419.96338.a3 19033739

[8] Rust H. Horizontal violence among nurses working in intensive care environments within the private healthcare sector: Stellenbosch. Stellenbosch University; 2018.

[9] Charati FG, Esmaeili R, Nasab NM, Jafari H. Occupational violence and its association with the quality of working life of nurses in intensive care units of educational-medical centers in 2019. Iran J Psychiatry Behav Sci. 2021;15(4):e114080.

[10] Habibzadeh H, Namadi F, Mahdi-Akhgar M. Nurse‐to‐nurse horizontal violence during the covid‐19 pandemic and its impact on turnover intention: A cross‐sectional study. Nurs open. 2023;10(4):2631–7. 10.1002/nop2.1523 PMC9877643 36463430

[11] Tlhako N, Coetzee SK, Ajanaku OJ, Fourie E. The impact of workplace relationships on nurse-reported quality of care and patient safety in the North West Province. PLoS ONE. 2025;20(5):e0323620. 10.1371/journal.pone.0323620 PMC12094723 40397912

[12] Bae SH. Noneconomic and economic impacts of nurse turnover in hospitals: A systematic review. Int Nurs Rev. 2022;69(3):392–404. 10.1111/inr.12769 PMC9545246 35654041

[13] Dumont C, Meisinger S, Whitacre MJ, Corbin G. Nursing2012 horizontal violence survey report. Nursing2025. 2012;42(1):44–9. 10.1097/01.NURSE.0000408487.95400.92 22157835

[14] Khalil D, editor. Levels of violence among nurses in Cape Town public hospitals. Nursing forum. Wiley Online Library; 2009. 10.1111/j.1744-6198.2009.00144.x 19691657

[15] Wilson BL, Diedrich A, Phelps CL, Choi M. Bullies at work: The impact of horizontal hostility in the hospital setting and intent to leave. JONA: J Nurs Adm. 2011;41(11):453–8. 10.1097/NNA.0b013e3182346e90 22033314

[16] Jung HS, Yoon HH. The effects of organizational service orientation on person–organization fit and turnover intent. Serv Ind J. 2013;33(1):7–29.

[17] Al-Nawafleh AH, Al-Hamdan ZM, Bawayzah H, Bawadi H. The influence of horizontal violence on intention to leave among Jordanian nurses: A cross-sectional study. PLoS ONE. 2024;19(11):e0307799. 10.1371/journal.pone.0307799 PMC11554039 39527610

[18] Zhang Y, Cai J, Yin R, Qin S, Wang H, Shi X, et al. Prevalence of lateral violence in nurse workplace: a systematic review and meta-analysis. BMJ open. 2022;12(3):e054014. 10.1136/bmjopen-2021-054014 PMC8966576 35351708

[19] Xu H, Xue M, Takashi E, Kitayama A, Zhu P, Liu Y. Exploring the relationship between lateral violence and nursing professionalism through the mediating effect of professional identity: A cross-sectional questionnaire study. Nurs open. 2023;10(5):3113–21. 10.1002/nop2.1561 PMC10077382 36572647

[20] Mayenti F, Meri D. Analysis lateral violance of nurses in accredited hospital in riau province.

[21] Peng X, Gan Y, Zeng Q, Xiong L, Zhang F, Xiong H, et al. Nurse-to‐nurse horizontal violence in Chinese hospitals and the protective role of head nurse’s caring and nurses’ group behaviour on it: A cross‐sectional study. J Nurs Adm Manag. 2022;30(6):1590–9. 10.1111/jonm.13498 PMC9787125 34699090

[22] Zhang Y, Yin R, Lu J, Cai J, Wang H, Shi X, et al. Association between horizontal violence and turnover intention in nurses: A systematic review and meta-analysis. Front public health. 2022;10:964629. 10.3389/fpubh.2022.964629 PMC9583538 36276344

[23] Pina D, Vidal-Alves M, Puente-López E, Luna-Maldonado A, Luna Ruiz-Cabello A, Magalhães T, et al. Profiles of lateral violence in nursing personnel of the Spanish public health system. PLoS ONE. 2022;17(5):e0268636. 10.1371/journal.pone.0268636 PMC9140284 35622880

[24] Vidal-Alves MJ, Pina D, Puente-López E, Luna-Maldonado A, Luna Ruiz-Cabello A, Magalhães T, et al. Tough love lessons: lateral violence among hospital nurses. Int J Environ Res Public Health. 2021;18(17):9183. 10.3390/ijerph18179183 PMC8431196 34501771

